# Rethinking the nonprofit foundation: an emerging niche in the rare disease ecosystem

**DOI:** 10.15252/emmm.201708203

**Published:** 2017-07-25

**Authors:** Annette C Bakker, Salvatore La Rosa

**Affiliations:** ^1^ Children's Tumor Foundation New York NY USA

**Keywords:** Cancer

## Abstract

In recent years, medical foundations have become increasingly influential, and now play an instrumental and integral role in the research and development of their disease area of interest. While some foundations have directly invested in taking drug candidates to the clinic, others have focused on creating specific tools for accelerating the identification and development of effective treatments. Here, we describe a new model, developed by the Children's Tumor Foundation (CTF), by which foundations may play a role in the rare disease ecosystem. On the one hand, the CTF uses its position to build bridges between academic scientists, biotech and pharmaceutical companies, and patients, to accelerate the development of treatments that really matter most to patients. On the other hand, it acts as a niche investor to fund an integrated platform for critical R&D endeavors—including elements such as a patient registry, biobank, and open data platforms—which smoothen the transition from basic discovery to clinical benefit. Currently, the Children's Tumor Foundation is launching a call to top finance experts to collaborate on building an innovative model that will guarantee long‐term sustainability of this integrated platform (Fig 1).

**Figure 1:**
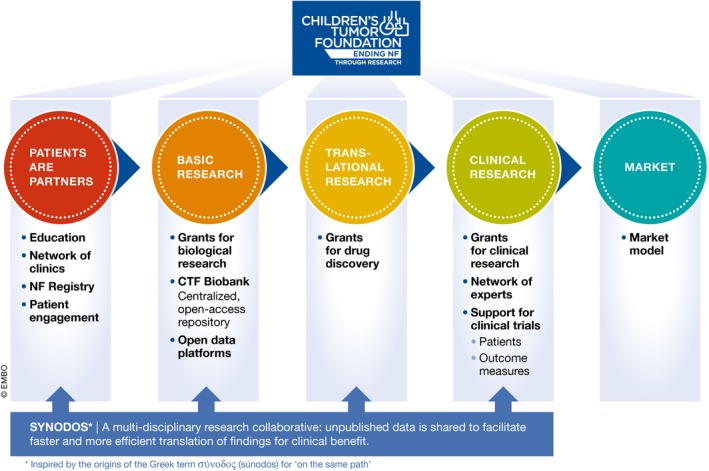
Overview of the initiatives of the Children's Tumor Foundation for accelerating drug discovery and development (R&D)

Medical philanthropies have raised and distributed billion dollars in research funding. Historically, their funding has been almost exclusively directed to grants that fund investigator‐initiated studies at academic research centers. The foundations publicized their impact by citing metrics, such as the number of grants funded, the number of publications generated, the number of researchers that were attracted to the field, and the amount of follow‐up support by governmental funding agencies. During the past few years, the overall landscape of philanthropic funding has drastically changed. A growing number of innovative foundations are no longer merely distributors of grant money, but have reinvented themselves as nonprofit enterprises with new, creative business models. This shift might also reflect a change in the staffing of medical nonprofit foundations from grant managers to experienced pharma and biotech executives.

As a consequence, foundations now play an instrumental role in the research and development process of their disease area of interest. The number of nonprofits that play a critical role in getting drug candidates to the market is rapidly growing. One highly successful example is the Cystic Fibrosis Foundation. It has taken a drug through clinical tests to market approval, which generates an impressive return on investment for the foundation (https://www.bloomberg.com/news/features/2015-07-07/this-medical-charity-made-3-3-billion-from-a-single-pill).

Another great success story is the development of Strimvelis, the first approved *ex vivo* gene therapy in Europe for severe combined immunodeficiency ADA‐SCID, by the Italian philanthropy Telethon and GlaxoSmithKline. Telethon, the San Raffaele Scientific Institute in Milano, and the joint San Raffaele Telethon Institute for Gene Therapy funded and organized research to cure this rare and severe disease. Along with GSK, they developed Strimvelis, which was approved for marketing in Europe in 2016 (Monaco & Faccio, 2017).

The Juvenile Diabetes Research Foundation (JDRF, http://www.jdrf.org/about/t1dfund/), as well as others, has started venture philanthropy arms that allow them to invest in early, risky projects without guarantee of a return on investment. Venture philanthropy is now playing a critical role in closing the gap between risky but crucial early‐stage research projects and mature but low‐risk/high‐return venture capital‐funded projects.

The common theme among these examples is that many foundations now invest directly into drug candidates or therapies. This more active role requires substantial financial means to support a balanced portfolio of both higher risk and lower risk projects. Moreover, there is no guarantee that investments into a specific drug candidate will yield any payback, or it will only occur years after the investment. This is especially risky for developing drugs for the up to 7,000 rare diseases with very distinct biological pathways. Exploring each rare disease would be extremely costly. It is therefore understandable that pharmaceutical and biotechnology corporations to date have not heavily invested in many rare diseases, including neurofibromatosis (NF), the focus of the Children's Tumor Foundation (CTF). However, since the pharmaceutical and biotech industries are essential for R&D and for moving new treatments to the market, the CTF has built a new business model to also attract these stakeholders to help find new treatments for NF.

The model is based on various observations. First, resources for R&D, such as a patient registry (Fig 2), biobanks, clinic networks, and open data and preclinical platforms, are often outside the scope of traditional funding agencies and philanthropies, but are absolutely essential to accelerate the drug discovery process. Second, foundations are often narrowly focused on their niche disease of interest. They are therefore very well connected with the specific research and patient community and can act as an efficient catalyst and project manager of collaboration. Third, financial support for research on rare diseases is very limited, and foundations should therefore not duplicate the funding from existing government and nongovernment funders. We discovered this to be true because, after having assessed the funding landscape, we discovered that: 1. The US National Institutes of Health (NIH) is committed to funding excellent science in a disease‐agnostic investigator‐initiated manner. This means the disease area may not see consistent levels of funding from year to year. 2. If the disease area can get support from other federal funding mechanisms such as the Congressionally Directed Medical Research Program (CDMRP, http://cdmrp.army.mil/nfrp/), the research community can benefit from disease‐directed funding. However, the CDMRP budget is prone to variations based on the approved budget. 3. In contrast, philanthropies are sometimes limited if individual donors wish to support a specific researcher, clinician, or institution. However, multiple experts at various institutions are often needed to address complex disease problems. 4. Finally, Venture Capitalists invest in specific products. Yet, they are usually driven by near‐term gain and therefore might be more risk‐averse.

**Figure 2:**
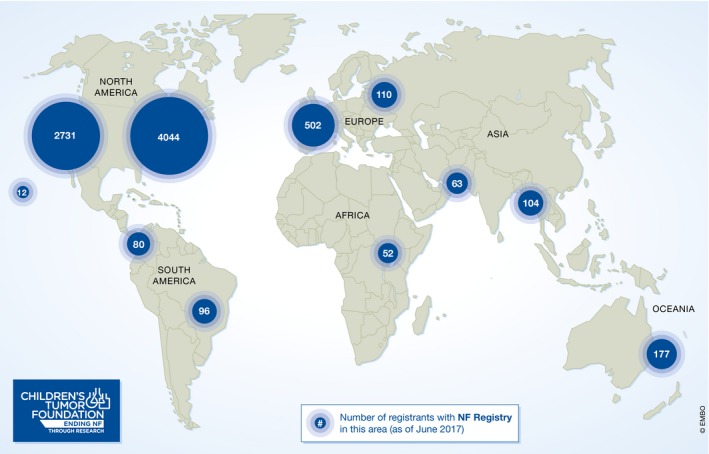
The number of patients that are enrolled in the NF patient registry that represents more than 8,000 patients from 98 countries (as of June 26, 2017)

The CTF has therefore decided to focus on two main opportunities: First, it has developed a new business model that allows our partners from the pharmaceutical and biotechnology industry to substantially accelerate their R&D process and to explore a rare disease, in this case neurofibromatosis, with almost no risk (Box 1). CTF's new “sandbox” model is an integrated research infrastructure that addresses challenges that are very prominent in rare disease, such as a small and scattered patient population; scarcity of tissue samples; scarcity of data and biological understanding; lack of tools, such as cell lines and animal models, to facilitate drug discovery; few experts in the field; lack of agreed outcome measures; and highly variable clinical care.

The one major challenge for this infrastructure is that the traditional fundraising model, relying on fundraising events and donors, is rather unpredictable, which contrasts with the need to guarantee long‐term sustainability of research resources. In this vein, the CTF is collaborating with multiple partners to develop a new, more stable financial model to consistently sustain the infrastructure.

Second, CTF has established a large collaborative multidisciplinary research consortium model that increases the efficiency of research and open data sharing. Based on its strong connection to the patient community and sharing the same sense of urgency as the patients, CTF saw the opportunity to strategically establish large collaborative research consortia that focus on disease‐specific research that matters most to patients. Inspired by the Stand Up to Cancer Initiative, the Foundation launched a NF “dream team” initiative called Synodos in 2014 (www.synapse.org/synodosNF2).

Synodos has the characteristics that we hope will accelerate research. First and foremost, the experience and requirements of the patients drive the topic of the research study. Only multidisciplinary teams—basic biology, translational science, and clinicians—may apply, and the co‐leaders, a clinician, and a researcher from the basic sciences are responsible for assembling the team and submitting a proposal. The Foundation organizes the review of the proposal by an international team of respected scientists who agree to stay on afterwards as reviewers and mentors of the freshly formed Synodos team. All Synodos members agree to share all raw data immediately with the NF data hub, which integrates and manages all the data. The project is managed by CTF scientists and driven by milestones, and payments are contingent upon the data sharing. Today the CTF manages five Synodos teams with 40 principal investigators from 20 different institutions.

Additionally, the CTF consortia model offers donors and funders, many of whom have a personal connection to NF, access to whole teams of researchers, rather than just individual scientists (if the funding is targeted to a single institution). This unique opportunity of gaining access to multiple research labs is also further enhanced by the fact that all data are immediately shared between the laboratories and, after a short embargo, with the global research community, and that experienced project managers employed by the CTF direct the project.

We have observed that many disease foundations have also taken a more proactive role in the current research environment. They have moved away from the classic investigator‐initiated award model toward taking an active stake in the discovery and development of specific treatments. The CTF has taken a slightly different approach. It is not yet investing in any specific drug candidates, but rather into the building of an infrastructure that reduces the risk for pharmaceutical companies to enter into neurofibromatosis as a disease area.

An additional benefit of this infrastructure is that in the long run, the entry barrier for industry is lowered, because they will not need to build the CTF‐created tools from scratch. This will hopefully give rise to new collaborative projects, new alliances, and alternative revenue streams from industry, which can be re‐directed toward the sustainability of the structure. The creation of specific, patient‐initiated programs such as the Synodos consortia also empowers our constituents to take a more proactive role—both as research participants and as donors, to direct their donations toward research that matters most to them.

Although the CTF model is focused on finding drug candidates for NF, we suggest that this business model can be applied to other rare diseases. Its scaling will depend on the availability of new, more predictable revenue models, thereby reducing the foundation's dependence on classical, unpredictable revenue streams.

**Box 1. The integrated infrastructures (“sandbox”) that CTF has invested in to accelerate drug discovery**

- The NF Registry (Seidlin *et al*, 2017) is a secure database that allows those living with NF to enter their own NF‐related medical information and data, and learn about clinical trials and initiatives connected to their particular manifestation of NF. It also allows CTF to help with the development of patient reported outcomes (PRO), and help pharmaceutical companies recruit patients for their clinical trials. The NF Registry currently hosts almost 8,000 patients and has been used approximately 20 times to help recruit patients for clinical trials.
- The NF Biobank (Von Dran *et al*, 2016) is a repository of patient tissue (such as blood and tumor samples) that is openly available to all researchers. The Foundation is also funding the full characterization of understudied tissue (Gosline *et al*, 2017) and the development of new cell lines for NF research and drug screening.
- Open NF data hub (www.synapse.org/CTF): All members of CTF research consortia are required to share every result, invention, new model, data, and new information generated within the consortium in real time with all members through the Synapse data portal provided by Sage Bionetworks. The data have an embargo of 12 months, and during this time, only the consortium members can access and use it. After this period, the Foundation has the contractual power to open the data to the public. At that time, the data can be accessed by all researchers and be used to launch new calls, public data challenges, hack‐a‐thons, or any other use for research purposes.
- A preclinical consortium is composed of multiple academic centers with continuously available NF GEM (Genetically Engineered Mouse) models to speed up drug testing.
- The program was launched by CTF almost 10 years ago. The platform is aimed at generating evidence of efficacy in NF animal models, using compounds already in the clinical stage of development for other indications (compound repurposing) in order to build a pipeline of compounds ready to enter clinical trials. This group has completed 116 preclinical trials that have informed 16 NF clinical trials.
- Importantly, the preclinical consortium has been instrumental in validating the involvement of MEK in plexiform neurofibroma growth. Their discoveries have led to the very promising phase 2 registration clinical trial (the effect of Selumetinib (AZD6244) on inoperable plexiform neurofibroma). AZD6244 shows in over 70% of the patients a tumor growth reduction of at least 20% (Jessen *et al*, 2013; Dombi *et al*, 2016).
- A network of Key Opinion Leaders (KOL) who act as a network of experts/consultants to speed up decision‐making and clinical trial design (Plotkin *et al*, 2013; Widemann & Plotkin, 2016; Blakeley *et al*, 2017).
- An international network of NF clinicians that define and agree on diagnostic, clinical care, and outcome measures for NF to allow a drug approval path (known as REiNS—Response Evaluation in NF and Schwannomatosis), as well as a national network of 50 NF clinics, which are connected to improve overall NF care (Merker *et al*, personal communication).

## Conflict of interest

Annette Bakker is the President and CSO of the Children's Tumor Foundation. Salvatore la Rosa is Vice President of Research of the Children's Tumor Foundation.
